# Integrated bioinformatics and machine learning identify S100A9 and VGLL1 as hub genes for schizophrenia

**DOI:** 10.3389/fpsyt.2025.1621219

**Published:** 2025-09-04

**Authors:** Jiankang Lv, Xueru Wang, Wei Qin

**Affiliations:** ^1^ Department of Severe Psychiatry, Shaoxing Seventh People's Hospital (Affiliated Mental Health Center, Medical College of Shaoxing University), Shaoxing, Zhejiang, China; ^2^ Department of Psychiatry, Shan Dong Daizhuang Hospital, Jining, Shandong, China; ^3^ Department of Psychiatry, Shandong Mental Health Center Affiliated to Shandong University, Jinan, Shandong, China

**Keywords:** schizophrenia, S100A9, VGLL1, bioinformatics analysis, machine learning

## Abstract

**Background:**

Schizophrenia (SCZ) is a debilitating neuropsychiatric disorder with unclear etiology, involving complex interactions between genetic and environmental factors. Current diagnostic methods rely on subjective clinical assessments, and existing treatments often fail to address cognitive and negative symptoms adequately. Identifying key biomarkers for SCZ is crucial for improving diagnosis and developing targeted therapies.

**Methods:**

This study integrated bioinformatics analysis and machine learning approaches to identify potential biomarkers for SCZ. Transcriptomic data from five independent cohorts were obtained from the GEO database. Differential expression analysis and Robust Rank Aggregation (RRA) were used to identify significant differentially expressed genes (DEGs). Protein-protein interaction (PPI) network, Least Absolute Shrinkage and Selection Operator (Lasso) regression and Random Forest (RF) were employed to screen for hub genes. The diagnostic model was constructed using logistic regression. The receiver operating characteristic (ROC) curve was used to evaluate diagnostic accuracy of the model, and nomograms and calibration curves were performed to evaluate their clinical applicability. Functional enrichment analyses and single-sample Gene Set Enrichment Analysis (ssGSEA) were conducted to explore the underlying mechanisms of the identified hub genes.

**Results:**

S100A9 and VGLL1 were determined as potential diagnostic biomarkers for SCZ. The diagnostic model demonstrated robust diagnostic performance in the training cohorts (AUC = 0.806) and external validation cohorts (AUC = 0.702, 0.666 and 0.739). Functional enrichment analyses revealed that DEGs related to VGLL1 and S100A9 were primarily involved in immune system regulation and signaling pathways such as PI3K-Akt signaling pathway. ssGSEA showed significant increases in the infiltration levels of five immune cell types (CD56bright natural killer cells, MDSCs, mast cells, natural killer cells, and plasmacytoid dendritic cells) in SCZ patients, with strong positive correlations between S100A9 and these immune cell infiltrations.

**Conclusion:**

Our study identified S100A9 and VGLL1 as potential biomarkers for SCZ, highlighting their roles in immune regulation. These findings provide new insights into the pathogenesis of SCZ and suggest potential diagnostic targets.

## 1 Introduction

Schizophrenia (SCZ) is a debilitating neuropsychiatric disorder affecting over 20 million individuals worldwide, characterized by a triad of positive symptoms (e.g., hallucinations), negative symptoms (e.g., social withdrawal), and cognitive dysfunction (1, 2). Despite its profound societal burden, the etiology of SCZ remains poorly understood. It is hypothesized that SCZ is associated with dysregulation of neurotransmission, defects in synaptic plasticity, and interactions between the nervous and immune systems (3). Current diagnosis relies on subjective clinical evaluations, while first-line antipsychotics (primarily targeting dopamine D2 receptors) exhibit variable efficacy and often fail to ameliorate cognitive or negative symptoms, accompanied by metabolic and extrapyramidal side effects (4). These limitations underscore the critical need for objective diagnostic tools and mechanism-based therapies.

The identification of biomarkers could bridge this gap by elucidating disease pathways and enabling targeted interventions. In oncology, biomarkers such as PD-L1 expression guide immunotherapy selection (5), while in neurodegenerative diseases, cerebrospinal fluid Aβ42/tau ratios aid Alzheimer’s diagnosis (6). In contrast, SCZ research faces a stark biomarker deficit. Although studies have proposed potential candidates (e.g., elevated IL-6 levels, hippocampal volume reduction, or polygenic risk scores) (7–9), none have achieved clinical validation due to heterogeneity across cohorts, low effect sizes, and poor reproducibility. This disparity highlights the urgency of discovering robust biomarkers specific to SCZ’s multifactorial pathology.

Emerging advances in machine learning provide powerful tools to decode complex biomarker patterns from high-dimensional omics data. Machine learning algorithms such as Least Absolute Shrinkage and Selection Operator (Lasso) regression and Random Forest (RF) have demonstrated success in other neuropsychiatric disorders (10). For example, Lasso-based models identified blood mRNA biomarkers predictive of major depressive disorder (11), while RF classifiers achieved >70% accuracy in distinguishing autism subtypes using metabolomic profiles (12). In addition, preliminary machine learning have linked gene co-expression networks to SCZ to stratify patient subgroups (13). This study aims to combine comprehensive bioinformatics analyses with machine learning to identify hub genes and molecular pathways from transcriptomic datasets. Our findings seek to unravel potential mechanisms underlying SCZ pathogenesis and propose novel biomarker candidates for diagnosis and therapeutic development.

## 2 Materials and methods

### 2.1 Data acquisition and integration

This study retrieved five SCZ-related microarray datasets (GSE12654, GSE21935, GSE17612, GSE53987, GSE38481) from the Gene Expression Omnibus (GEO) database, comprising a total of 224 brain tissue samples (112 SCZ and 112 controls) and 37 whole blood sample (22 SCZ and 15 controls). Detailed dataset information is provided in **Table 1**. Quantile normalization was performed using the “limma” package to eliminate technical variability, and the ComBat algorithm was applied to correct inter-platform batch effects. After quality control, GSE12654 and GSE21935 were merged as the training cohort, while GSE17612, GSE53987 and GSE38481 served as independent external validation cohorts. The effectiveness of data integration was validated using boxplots and principal component analysis (PCA) generated by ggplot2.

**Table 1:** Detailed information of GEO datasets.

Datasets	Platforms	Sample source	Control	SCZ	Type
GSE12654	GPL8300	prefrontal cortex (BA10)	15	13	array
GSE21935	GPL570	temporal cortex (BA22)	19	23	array
GSE17612	GPL570	prefrontal cortex (BA10)	23	28	array
GSE53987	GPL570	Hippocampus Pre-frontal cortex (BA46) Associative striatum	55	48	array
GSE38481	GPL6883	Whole blood	22	15	array

### 2.2 Identification of differentially expressed genes

Differential expression analysis was conducted on the training cohort, GSE12654 and GSE21935 using the “limma” package, with screening criteria set at p-value <0.05 and |logFC| > 0.585 (14, 15). Heatmap and volcano plots of DEGs were generated using “pheatmap” and “ggplot2” packages, respectively.

### 2.3 Robust rank aggregation analysis

The RRA algorithm integrates gene ranking information across datasets via a probabilistic model to identify consistently significant DEGs across multiple independent datasets. In this study, RRA was applied to rank up- and down-regulated DEGs from all datasets based on logFC. Aggregated ranking scores were used to compute p-value, and genes with p-value <0.05 and |logFC| > 0.585 were selected as DEGs. RRA analysis was implemented using the “RobustRankAggreg” package.

### 2.4 Enrichment analysis

Gene Ontology (GO) and Kyoto Encyclopedia of Genes and Genomes (KEGG) pathway enrichment analyses were performed using the “clusterProfiler” and “org.Hs.eg.db” packages. Significantly enriched GO terms and KEGG pathways were defined as those with p-value <0.05.

### 2.5 Protein-protein interaction network

PPI networks of DEGs were constructed using the STRING database and visualized with Cytoscape software. Feature DEGs were identified using 10 topological network algorithms (MCC, MNC, etc) via the cytoHubba plugin.

### 2.6 Machine learning

Lasso regression and RF were employed for hub genes selection. Lasso regression is a linear regression method used for feature selection and sparse modeling. It incorporates an L1 regularization term into the objective function to select fewer features, thereby reducing the risk of overfitting. In this study, Lasso regression was implemented using the “glmnet” package with the following parameters: family = binomial, type.measure = class, alpha = 1, and nfold = 10. RF, an ensemble learning method based on decision trees, was employed to capture non-linear relationships and assess feature importance. By constructing multiple decision trees and combining their predictions, RF enhances model accuracy and controls overfitting. The RF algorithm was performed using the “randomForest” package with ntree = 500. Feature importance was evaluated using the Gini coefficient, with a threshold of >2 used for selecting features DEGs.

### 2.7 Construction and validation of the diagnostic model

We utilized the “glm” function from the “glmnet” package to construct a logistic regression model. This function is a standard tool for fitting generalized linear models, particularly suitable for logistic regression analysis in binary classification problems. The expression level of feature DEGs was severed as the independent variable, with the disease diagnosis outcome (SCZ = 1, Control = 0) as the dependent variable. The parameter was set as family = binomial (link=‘logit’). The formula of the model: y= β_0_+β_1_*X_1_+ β _2_*X_2_+ β _3_*X_3_+⋯+β_i_*X_i_ (β was coefficient, X was the expression level of genes). Diagnostic performance was evaluated via receiver operating characteristic (ROC) curve in the training cohort and external validation cohorts. The nomogram for predicting disease risk was constructed using the “rms” package, and the clinical applicability of the model was assessed through a calibration curve.

### 2.8 Immune infiltration analysis

single-sample gene set enrichment analysis (ssGSEA) quantified infiltration levels of 28 immune cell subtypes (16, 17). Wilcoxon rank-sum test was used to compare immune cell infiltration between SCZ patients and controls. Spearman’s rank correlation analyzed associations between hub genes and immune cells.

## 3 Results

### 3.1 Identification of DEGs

**Figure 1:**
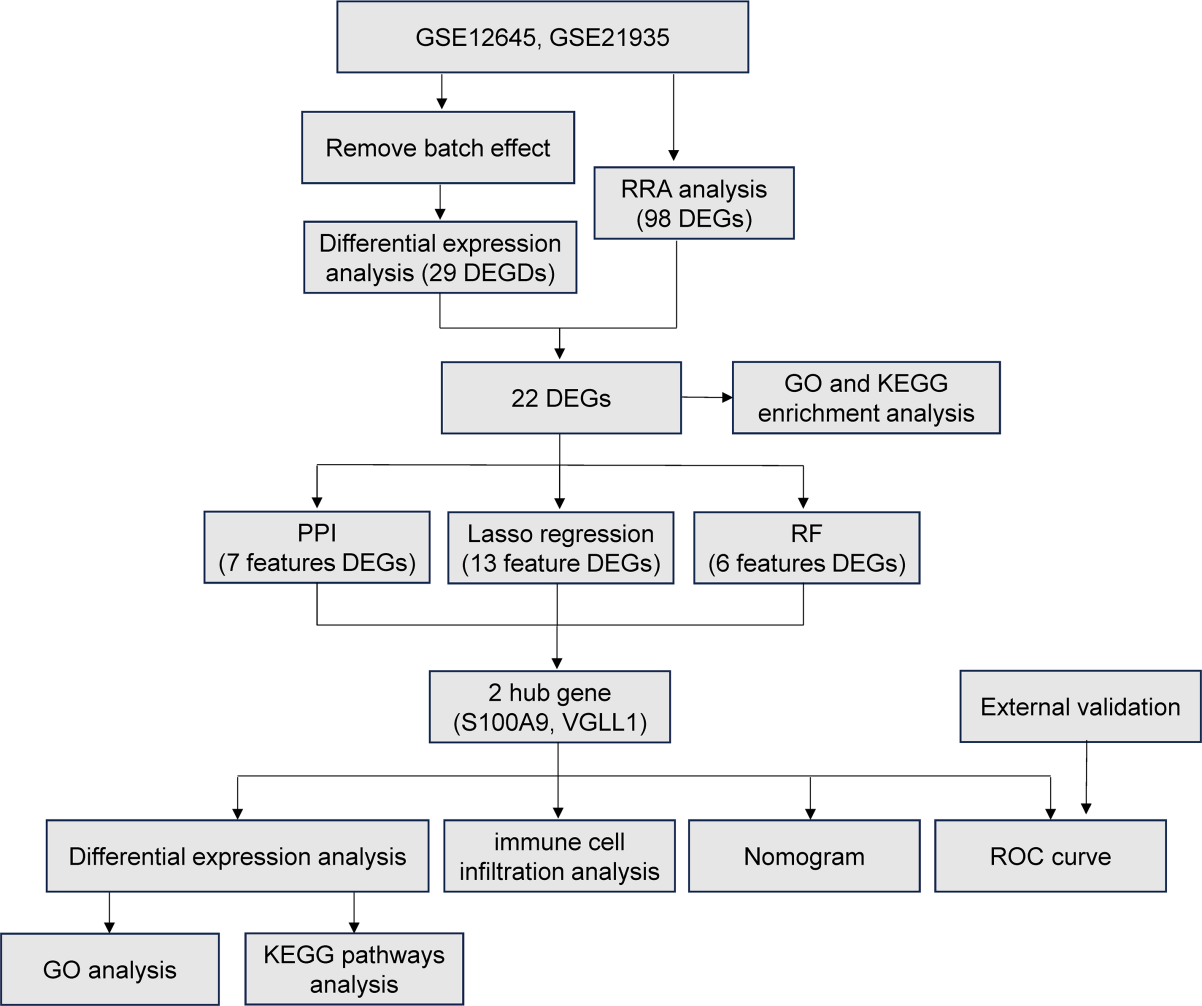
The study workflow.

**Figure 2:**
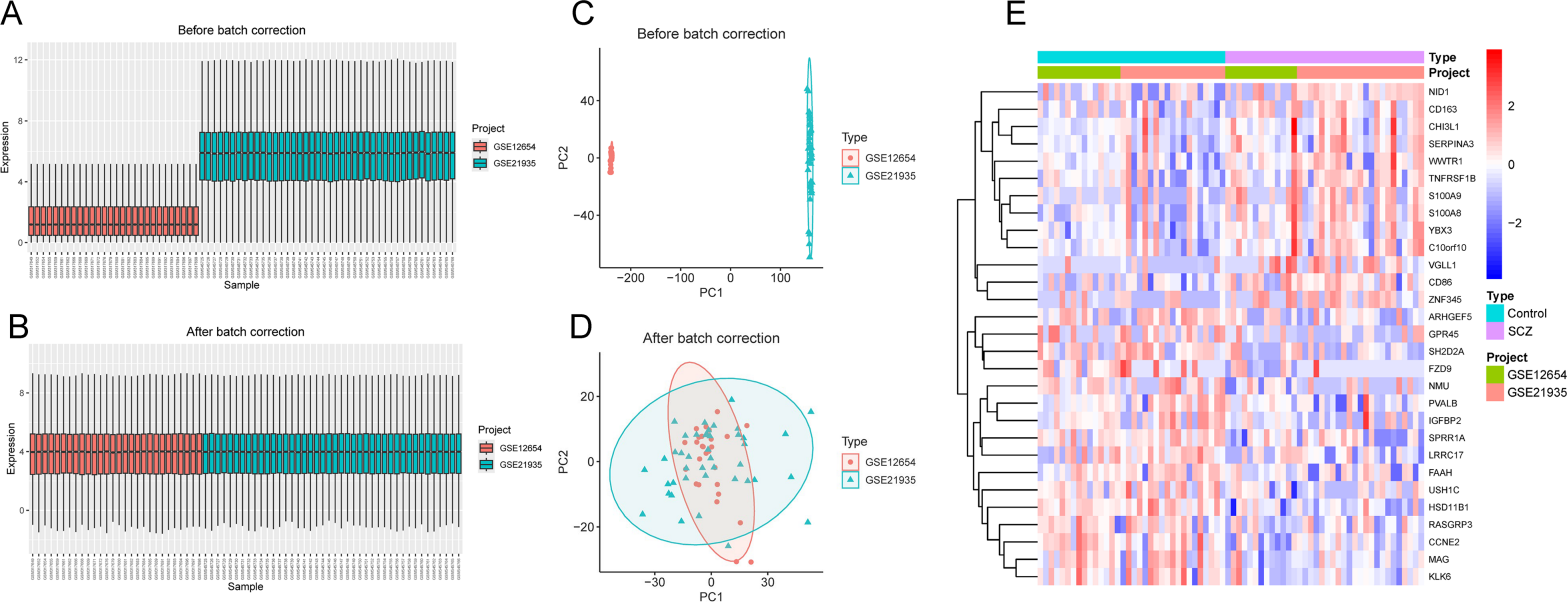
Batch effect correction. **(A, B)** Boxplots of gene expression distributions before **(A)** and after **(B)** batch effect correction using the ComBat algorithm. **(C, D)** PCA before **(C)** and after **(D)** batch correction using the ComBat algorithm. **(E)** Heatmap of 29 DEGs in the training cohort.

**Figure 3:**
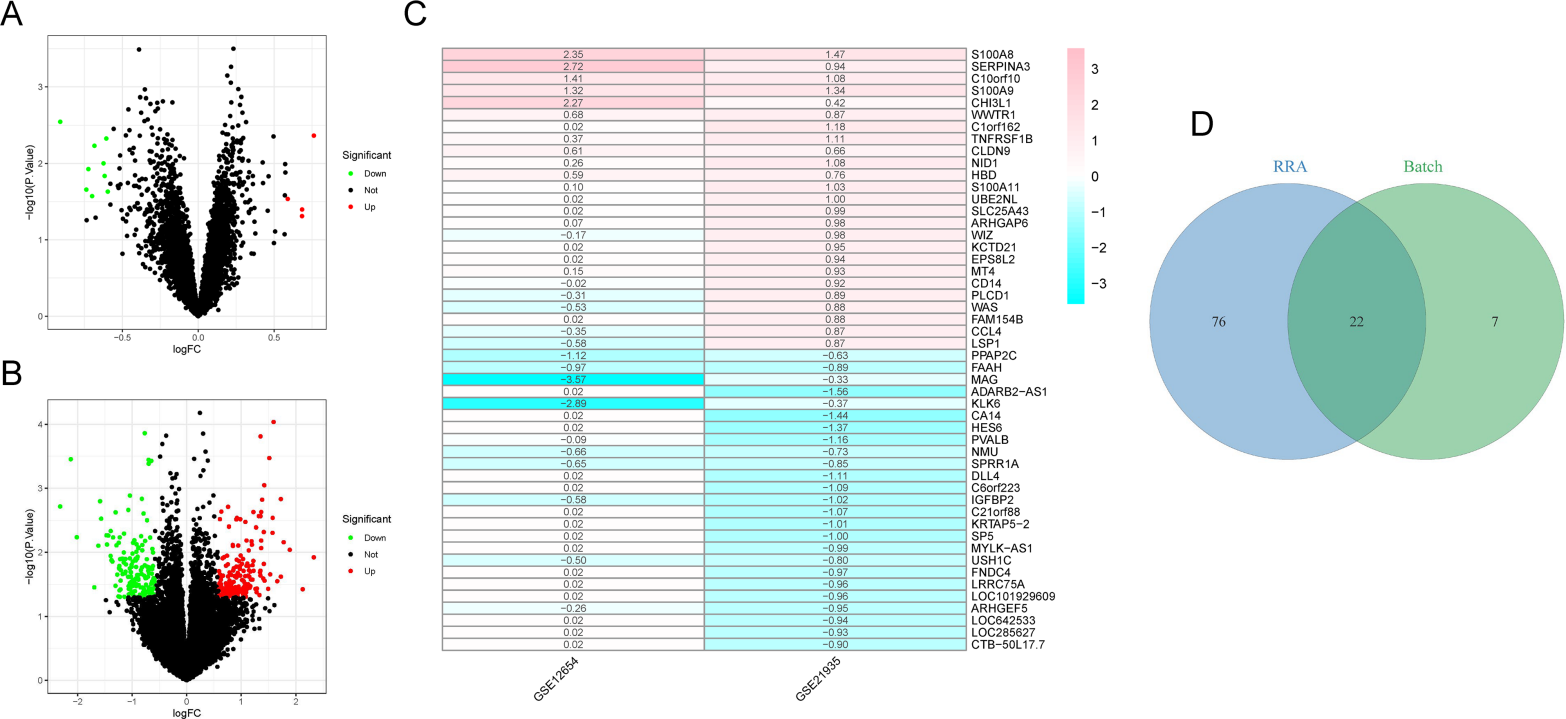
RRA analysis. **(A, B)** Volcano plots of DEGs in GSE12654 and GSE21935 dataset. Red and green dots represent upregulated and downregulated DEGs, respectively. **(C)** Heatmap of 50 DEGs (25 upregulated and 25 downregulated) identified through RRA analysis. **(D)** Venn diagram showing the intersection of DEGs from the training cohort and RRA analysis.

The study workflow was illustrated in **Figure 1**. After batch effect correction, showed significant improvements in sample clustering (**Figures 2A-D**). In the training cohort, a total of 29 DEGs were identified, including 16 downregulated and 13 upregulated genes (**Figure 2E**, **Supplementary Table S1**). Before RRA analysis, differential expression analyses were conducted separately on the GSE12654 and GSE21935 datasets, and the results were shown by volcano plots (**Figures 3A, B**). After integration using the RRA algorithm, a total of 98 DEGs were identified, comprising 41 downregulated and 57 upregulated genes (**Figure 3C**, **Supplementary Table S2**). Taking the intersection of these DEGs, we ultimately identified 22 significant DEGs (**Figure 3D**).

### 3.2 Enrichment analysis of DEGs

**Figure 4:**
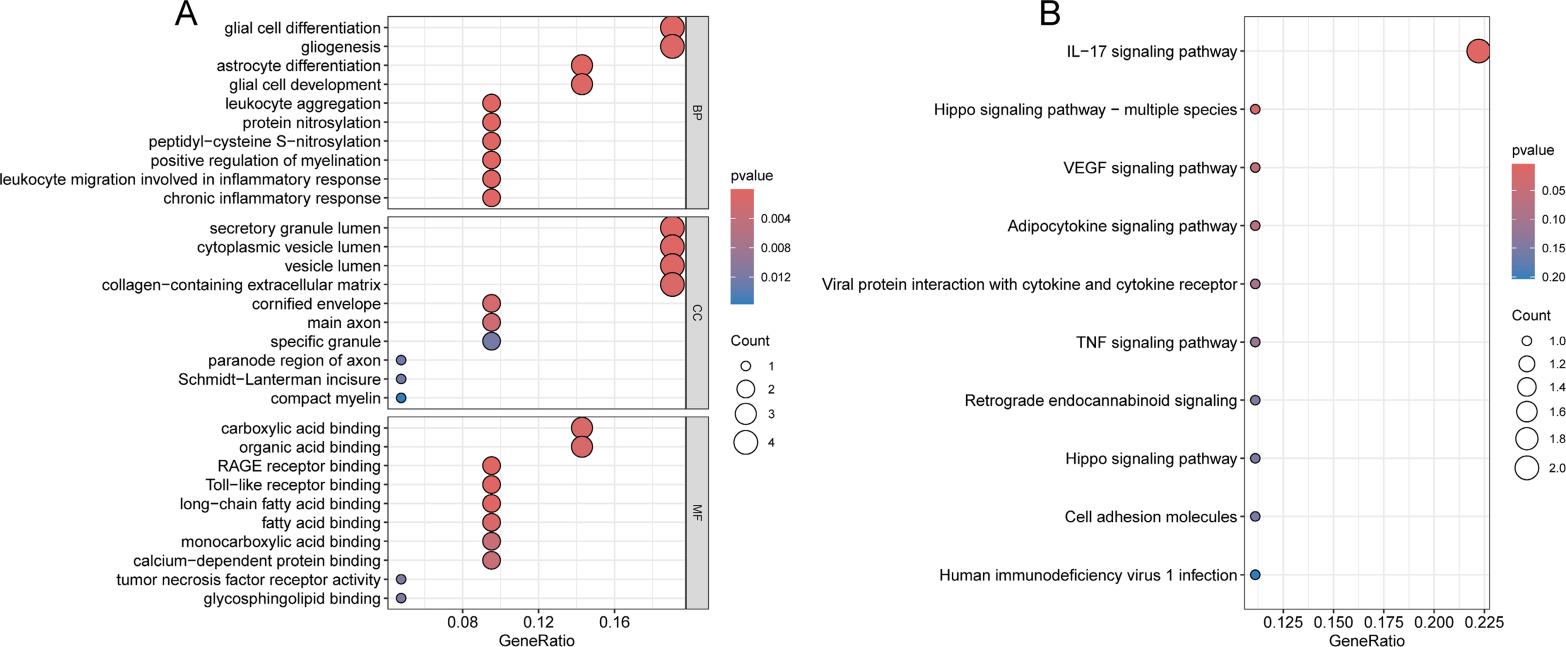
Functional enrichment analysis of 22 DEGs. **(A)** GO analysis of 22 DEGs. **(B)** KEGG pathways analysis of 22 DEGs.

Subsequently, GO and KEGG enrichment analyses were performed on the 22 significant DEGs. The biological processes (BP) with high significance were related to nervous system development and function, including glial cell differentiation, astrocyte differentiation; Cellular component (CC) showed significant enrichment of DEGs in secretory granule lumen, collagen−containing extracellular matrix; Molecular function (MF) revealed significant enrichment of DEGs in carboxylic acid binding and organic acid binding (**Figure 4A**). KEGG pathway analysis further indicated that DEGs were enriched in several key signaling pathways involved in biological processes, such as IL-17, TNF and Hippo signaling pathways (**Figure 4B**).

### 3.3 Identification of hub genes

**Figure 5:**
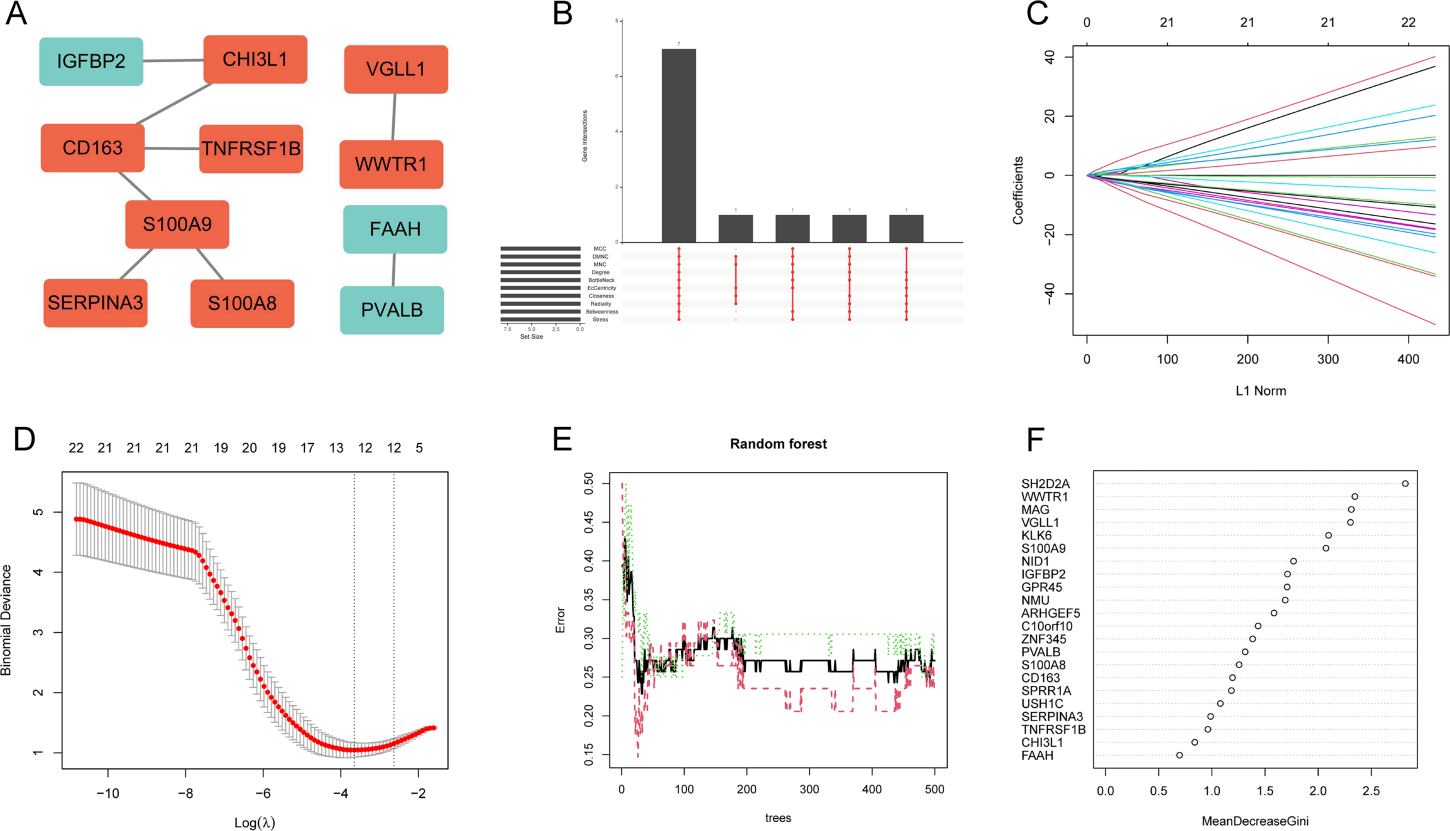
Identification of hub genes using PPI networks and machine learning. **(A)** PPI network of DEGs. **(B)** Upset of the top 10 DEGs from 10 topological network algorithms. **(C, D)** Lasso regression to identify 13 feature DEGs. **(E, F)** RF algorithm selected 6 feature DEGs.

**Figure 6:**
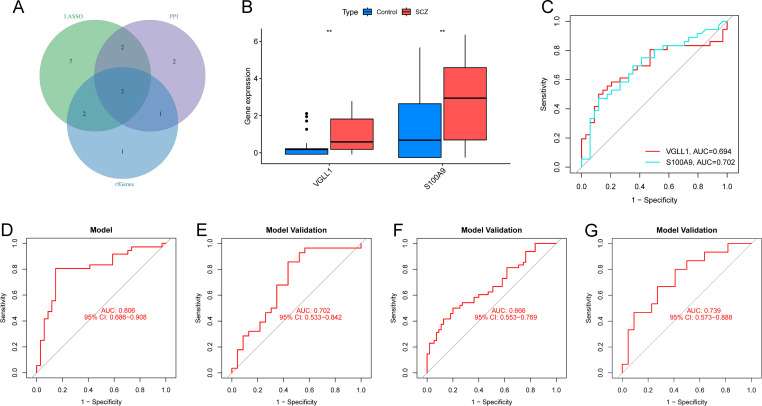
Identification and validation of the diagnostic model. **(A)** Intersection of feature DEGs from PPI network, lasso regression and RF algorithm. **(B)** Boxplots showing the differential expression of S100A9 and VGLL1 in training cohort. ** p-value <0.01. **(C)** ROC curves for individual genes in training cohort. **(D)** ROC curve for diagnostic model in training cohort. **(E–G)** External validation in GSE17612 **(E)**, GSE53987 **(F)** and GSE38481 **(G)**.

To systematically identify hub genes associated with disease pathogenesis, we first constructed PPI network to visualize the interactions among 22 DEGs. The results showed that 11 of the 22 DEGs had interactions (**Figure 5A**). Using 10 topological network algorithms to rank genes, the intersection of the top 10 genes from each algorithm was taken, with a total of 7 genes (S100A9, CHI3L1, WWTR1, VGLL1, SERPINA3, S100A8, PVALB) identified as feature DEGs (**Figure 5B**). Subsequently, lasso regression and RF were employed to identify feature DEGs. Lasso regression analysis selected 13 feature DEGs (MAG, VGLL1, S100A8, SPRR1A, ZNF345, S100A9, USH1C, NMU, SH2D2A, GPR45, SERPINA3, ARHGEF5, IGFBP2) (**Figures 5C, D**). while RF identified six feature DEGs (SH2D2A, WWTR1, MAG, VGLL1, KLK6, and S100A9) with Gini coefficients >2 (**Figures 5E, F**). Ultimately, through the intersection analysis of these features DEG subsets, two DEGs (S100A9 and VGLL1) were determined as the optimal hub genes for SCZ (**Figure 6A**).

### 3.4 Construction and validation of the diagnostic model

**Figure 7:**
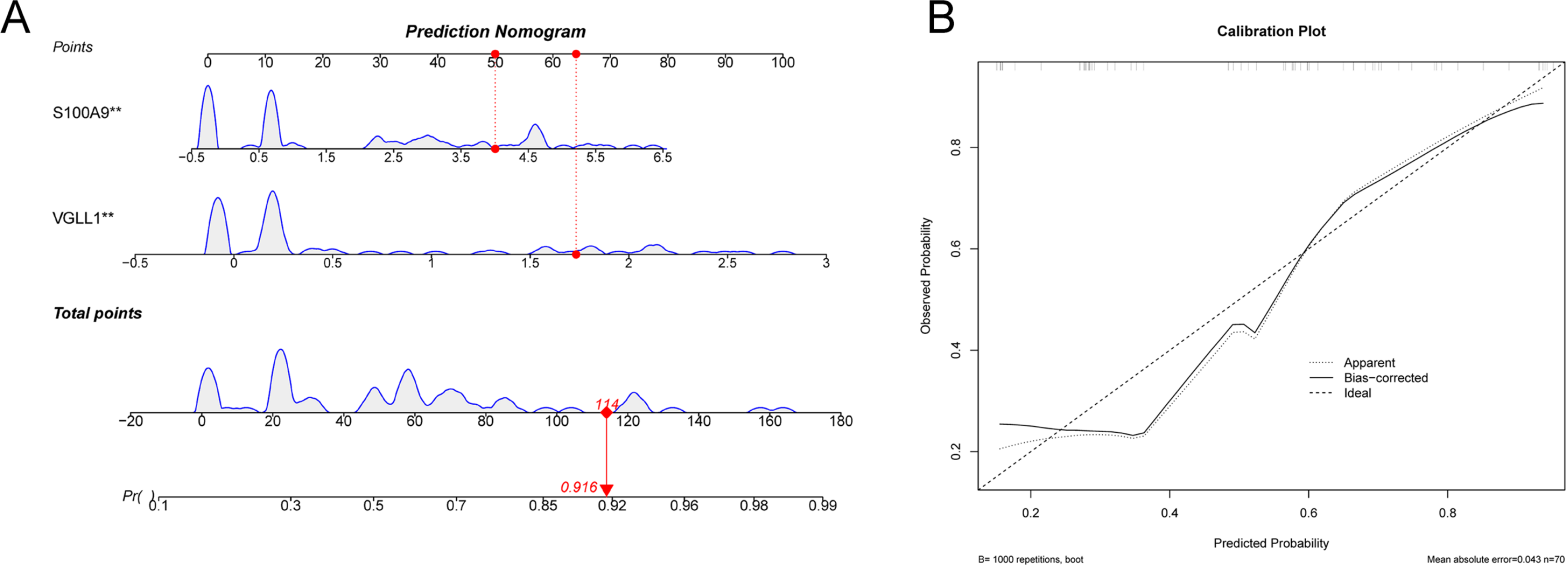
Nomogram and calibration curves for the diagnostic model. **(A)** The nomogram was used to predict the incidence of SCZ. **(B)** The calibration curve for evaluated the predictive power of the model.

The diagnostic value of the hub genes in SCZ was further evaluated. In the training cohort, S100A9 and VGLL1 were significantly upregulated in SCZ groups (**Figure 6B**). The area under curve (AUC) values of ROC curve for these genes was 0.702 and 0.694, respectively (**Figure 6C**), indicating their diagnostic potential. Subsequently, a logistic diagnostic model was constructed based on the expression levels of S100A9 and VGLL1, with the formula: y=−1.4815 + 1.2469×VGLL1 + 0.4252×S100A9. The model achieved an AUC of 0.806 in the training cohort, demonstrating good discriminatory ability for SCZ (**Figure 6D**). Subsequently, we performed external validation of the predictive model’s discriminative performance using gene expression profiles of independent validation cohorts (GSE17612, GSE53987, and GSE38481). The results demonstrated that the model achieved AUC values of 0.702, 0.666, and 0.739 in the three external cohorts, respectively, further confirming its diagnostic efficacy and generalizability across independent datasets (**Figures 6E-G**). Additionally, we constructed a nomogram to predict the risk of SCZ (**Figure 7A**), and calibration curve analysis showed high consistency between predicted and actual SCZ risks (**Figure 7B**).

### 3.5 S100A9-related DEGs and functional enrichment analysis

**Figure 8:**
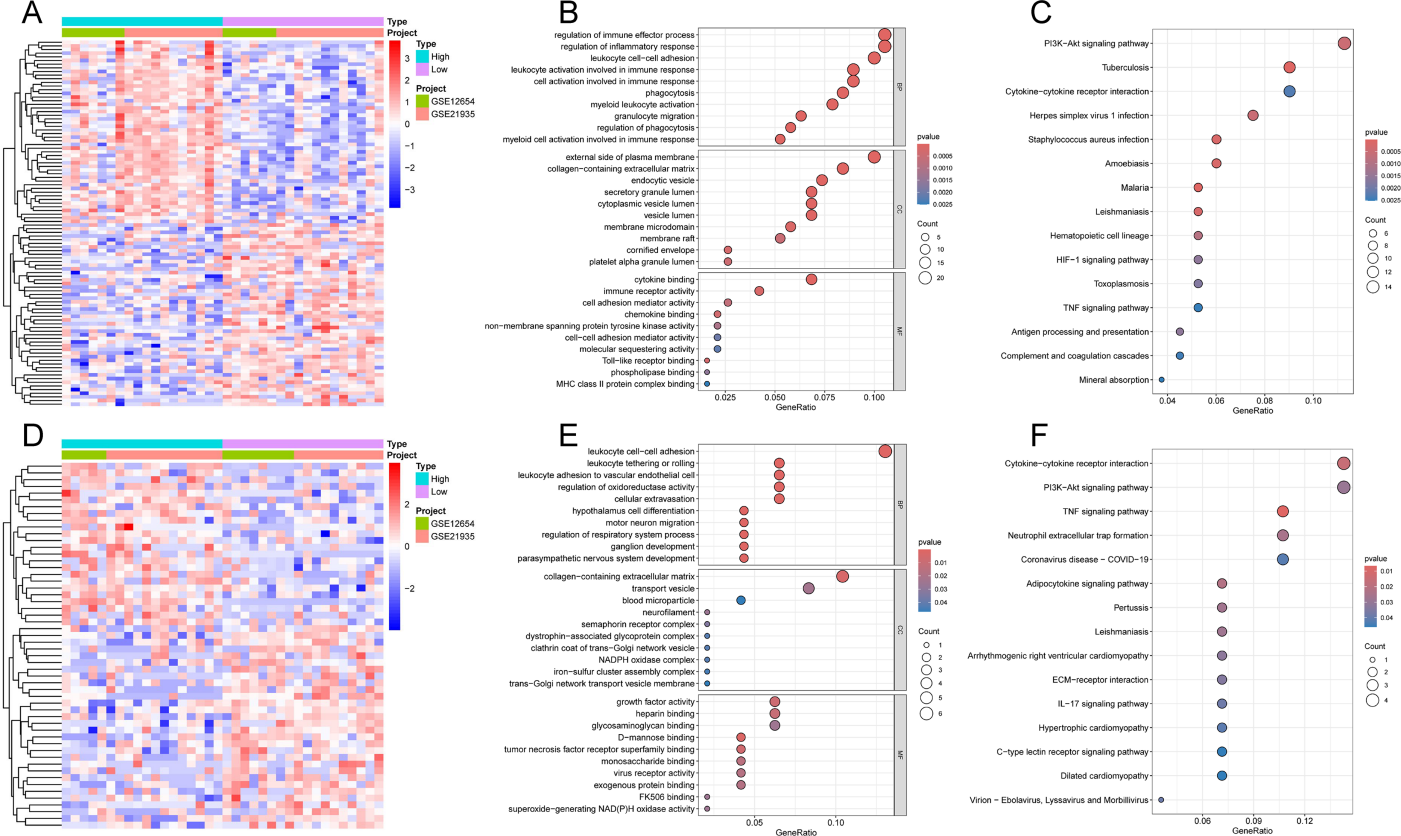
Functional enrichment analysis of hub-related DEGs. **(A)** Heatmap of S100A9-related DEGs. **(B)** GO analysis of S100A9-related DEGs. **(C)** KEGG pathways analysis of S100A9-related DEGs. **(D)** Heatmap of VGLL1-related DEGs. **(E)** GO analysis of VGLL1-related DEGs. **(F)** KEGG pathways analysis of VGLL1-related DEGs.

To elucidate the potential molecular mechanisms of hub genes in the development of SCZ, we divided the SCZ samples in the training cohort into high- and low-expression groups based on the median expression of the hub genes and performed differential expression and functional enrichment analyses. Based on the median expression of S100A9, a total of 211 DEGs were identified, including 67 downregulated and 144 upregulated genes (**Figure 8A**, **Supplementary Table S3**). GO analysis (**Figure 8B**) indicated that S100A9-related DEGs were primarily involved in BP related to immune system regulation, such as regulation of immune effector process, leukocyte cell−cell adhesion, and cell activation involved in immune response. In terms of CC, DEGs were significantly enriched in external side of the plasma membrane and collagen−containing extracellular matrix. Regarding MF, DEGs were significantly enriched in cytokine binding, immune receptor activity, and cell adhesion mediator activity. Additionally, KEGG pathway analysis showed that DEGs were primarily involved in the PI3K-Akt, HIF-1, and TNF signaling pathways (**Figure 8C**).

### 3.6 VGLL1-related DEGs and functional enrichment analysis

Based on the median expression of VGLL1, a total of 54 DEGs were identified, including 30 downregulated and 24 upregulated genes (**Figure 8D**, **Supplementary Table S4**). GO analysis showed that the main BP enriched by DEGs were also related to the immune system, such as leukocyte cell-cell adhesion and leukocyte adhesion to vascular endothelial cells; in addition, collagen-containing extracellular matrix, transport vesicles, growth factor activity, and heparin binding were significantly enriched in CC and MF (**Figure 8E**). KEGG pathway analysis further revealed that DEGs were significantly enriched in PI3K-Akt, TNF and IL-17 signaling pathways (**Figure 8F**). These results, similar to those of S100A9-related DEGs, suggest the important regulatory roles of the immune system and signaling pathways in the pathogenesis of SCZ.

### 3.7 Immune infiltration analysis

**Figure 9:**
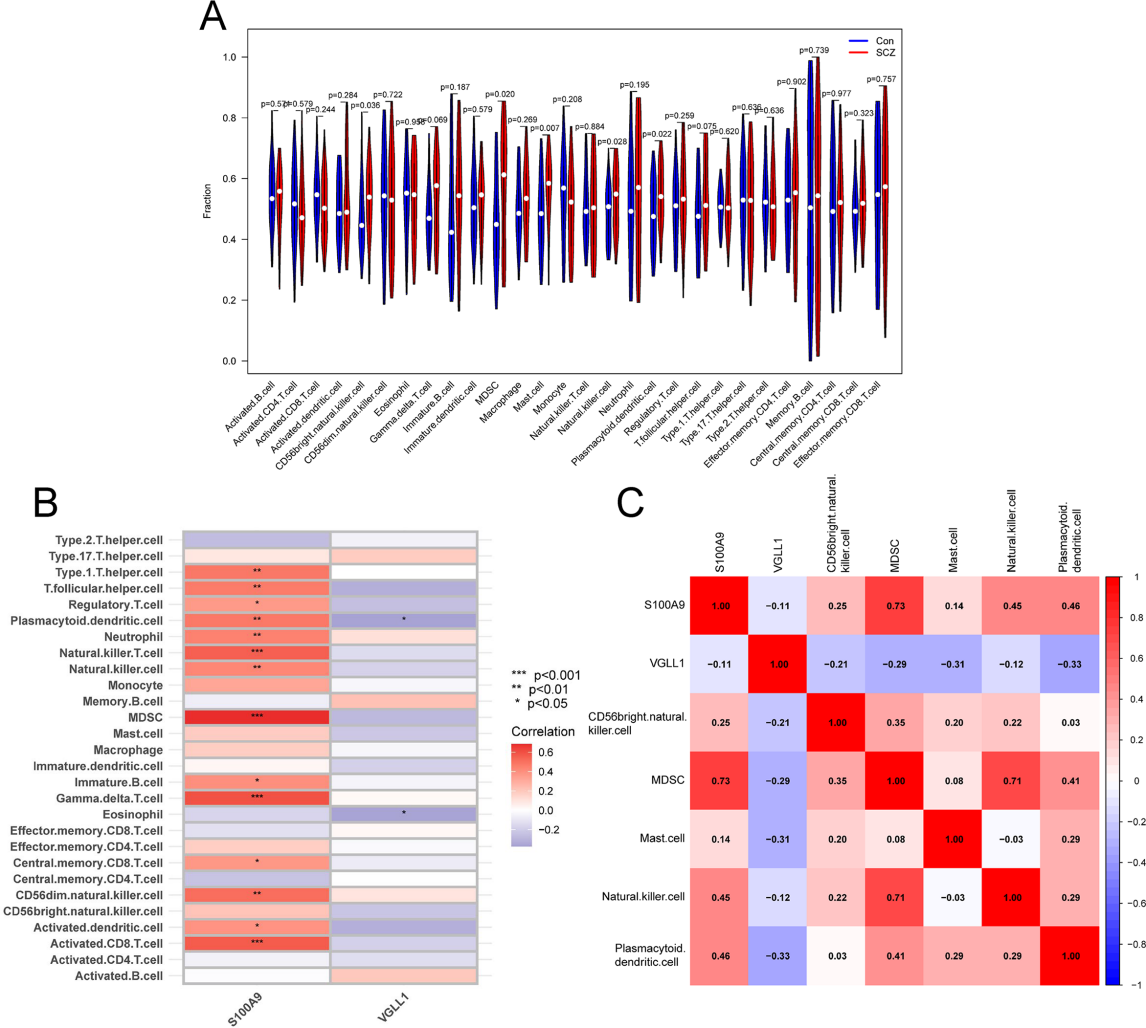
Immune infiltration analysis. **(A)** The violin plot showing the differences in immune infiltrating of 28 immune cell subtypes between SCZ and control groups. **(B)** Correlation between hub genes and 28 immune cell subtypes. **(C)** Correlation between hub genes and five significantly infiltrating immune cells.

Given the enrichment analysis results indicating the involvement of hub genes in immune system responses, we further employed ssGSEA to analyze the infiltration levels of 28 immune cell types in the training cohort. The analysis revealed significant increases in the infiltration levels of five immune cell types in the SCZ group, including CD56bright natural killer cells, MDSCs, mast cells, natural killer cells, and plasmacytoid dendritic cells (**Figure 9A**). Correlation analysis further showed that S100A9 was significantly positively correlated with these immune cells, while VGLL1 was negatively correlated with plasmacytoid dendritic cells (**Figures 9B, C**). Collectively, our results suggest that hub genes may be involved in the pathogenesis of SCZ through the regulation of immune cell infiltration.

## 4 Discussion

SCZ, a complex neuropsychiatric disorder with an incompletely elucidated etiology, arises from intricate interactions between genetic predispositions and environmental factors (18). Emerging evidence highlights its association with dysregulated gene expression and immune system dysfunction (19). Although numerous studies have identified multiple genetic loci linked to SCZ, the critical genes driving its pathogenesis remain to be fully characterized (20). Given the substantial burden imposed by SCZ, the identification of novel diagnostic targets, coupled with exploration of the diversity and complexity of the immune microenvironment, is pivotal for achieving early diagnosis.

In this study, we employed integrated bioinformatics and machine learning to systematically screen SCZ-related biomarkers across multiple dimensions. Initially, differential expression analysis combined with RRA algorithm identified 22 significant DEGs. Subsequently the hub genes were further cross-identified through the PPI network and the RF and LASSO regression. Ultimately, we determined VGLL1 and S100A9 as potential diagnostic biomarkers for SCZ. A logistic regression model based on hub genes demonstrated good diagnostic performance in both the training cohort (AUC = 0.806) and external validation cohorts (AUC = 0.702 and 0.666), highlighting their clinical potential as SCZ biomarkers. Additionally, a nomogram based on the hub genes further demonstrated their potential for clinical application.

Subsequently, we explored the potential mechanisms of the hub genes in SCZ pathogenesis. Enrichment analysis revealed that S100A9- and VGLL1-related DEGs were primarily involved in immune system regulation and key signaling pathways, including the PI3K-Akt and TNF signaling pathway. ssGSEA showed significant increases in the infiltration levels of five immune cell types in SCZ patients, including CD56bright natural killer cells, MDSCs, mast cells, natural killer cells, and plasmacytoid dendritic cells. Notably, S100A9 exhibited strong positive correlations with the infiltration of these immune cells, while VGLL1 showed a negative correlation with plasmacytoid dendritic cells.

The S100 protein family, implicated in neuroinflammation and astrocyte activation, is recognized as a contributor to schizophrenia pathogenesis. S100 proteins are significantly upregulated in the brain tissue, blood, and other body fluids of SCZ patients (21). S100A9, a pro-inflammatory calcium-binding protein within this family, is involved in various intracellular and extracellular biological processes, including cell differentiation, inflammatory responses, immune regulation, and intercellular signaling (22, 23). Recent studies have highlighted the role of S100A9 in the nervous system, particularly in neuropsychiatric disorders (24, 25). A recent study revealed that S100A9 drove microglial hyperactivation via the TLR4/NF-kB pathway, correlating with elevated neuroinflammatory markers in the cerebrospinal fluid of SCZ patients (26, 27). As a marker for MDSCs, S100A9 modulated MDSC-mediated immune suppression by binding to TLR4 and RAGE (28, 29). CD56bright natural killer cells, a subset of natural killer cells primarily secreting cytokines -IFN-γ, exhibit increased percentages in acutely relapsed SCZ patients, potentially serving as a disease trait marker (30, 31). Additionally, research found that S100A9 enhanced IFN-γ production in NK cells via p38 MAPK pathway activation (32). Plasmacytoid dendritic cells are an important part of the immune system and are responsible for antigen presentation and cytokine secretion. Studies have shown that S100A9 was expressed on the surface of plasmacytoid dendritic cells, and when activated, S100A9 will be actively transported to the outside of the membrane, indicating that it may have biological functions (33). However, its exact role in plasmacytoid dendritic cells remains to be further confirmed. In summary, S100A9 may play an important role in immune regulation of schizophrenia through its interaction with multiple immune cells.

VGLL1, a transcriptional coactivator, regulates cell proliferation and differentiation by interacting with TEAD4, a transcription factor in the Hippo signaling pathway (34). Although its direct role in immune cells remains unclear, KEGG analysis indicated VGLL1-related DEGs were enriched in the PI3K-Akt signaling pathway, which is a crucial pathway for the activation and proliferation of various immune cells (35, 36). Thus, VGLL1 may modulate immune cell functions by influencing the PI3K-Akt signaling pathway. MDSCs suppress the functions of NK cells and T cells by secreting TGF-β and IL-10 (37), and VGLL1 may indirectly affect the immunosuppressive functions of MDSCs by regulating these cytokine interactions. While no studies have explicitly linked VGLL1 to SCZ mechanisms, our findings provide initial insights into this point. Future research should investigate the interactions between VGLL1 and immune regulation, as well as its role in the pathogenesis of SCZ, to determine whether it can serve as a potential target for diagnosis and treatment.

This study identified VGLL1 and S100A9 as novel diagnostic biomarkers for SCZ through integrated bioinformatics and machine learning, revealing their potential roles in disease progression through immune regulation. However, there are still some limitations. Firstly, potential limitations exist in the bioinformatics analytical methodology. For instance, data preprocessing approaches may introduce bias, as data selection and normalization procedures could lead to divergent analytical outcomes. Furthermore, the selected analytical tools (e.g., limma for differential expression analysis) have inherent limitations in their ability to fully capture the complexity of biological systems due to their predefined algorithms and assumptions. Second, all samples were derived from different public datasets, which may introduce heterogeneity in disease subtypes and clinical characteristics. This necessitates validation through independent clinical cohorts to ensure the robustness and generalizability of our findings. Thirdly, functional experiments are needed to elucidate the causal roles of the hub genes in SCZ. Finally, in future research, we could leverage single-cell sequencing to dissect the molecular interactions between specific brain regions and immune subpopulations, offering new directions for precision treatment of SCZ.

## Data Availability

The original contributions presented in the study are included in the article/**Supplementary Material**. Further inquiries can be directed to the corresponding author.

## Supplementary material

Supplementary Table 1 29 DEGs of the training cohort.

Supplementary Table 2 98 DEGs of RRA analysis.

Supplementary Table 3 211 S100A9-related DEGs.

Supplementary Table 4 54 VGLL1-related DEGs.

## References

[1] Carpenter WT Tandon R. Psychotic disorders in DSM-5: summary of changes. Asian J Psychiatr. (2013) 6:266–8. doi: 10.1016/j.ajp.2013.04.001, PMID: 23642992

[2] GMD C. Global, regional, and national burden of 12 mental disorders in 204 countries and territories, 1990-2019: a systematic analysis for the Global Burden of Disease Study 2019. Lancet Psychiatry. (2022) 9:137–50. doi: 10.1016/s2215-0366(21)00395-3, PMID: 35026139 PMC8776563

[3] Orrico-Sánchez A López-Lacort M Muñoz-Quiles C Sanfélix-Gimeno G Díez-Domingo J. Epidemiology of schizophrenia and its management over 8-years period using real-world data in Spain. BMC Psychiatry. (2020) 20:149. doi: 10.1186/s12888-020-02538-8, PMID: 32248839 PMC7132863

[4] Furiak NM Ascher-Svanum H Klein RW Smolen LJ Lawson AH Montgomery W. Cost-effectiveness of olanzapine long-acting injection in the treatment of patients with schizophrenia in the United States: a micro-simulation economic decision model. Curr Med Res Opin. (2011) 27:713–30. doi: 10.1185/03007995.2011.554533, PMID: 21265593

[5] Du S Liu J Zhang Y Ge X Gao S Song J. PD-L1 peptides in cancer immunoimaging and immunotherapy. J Control Release. (2025) 378:1061–79. doi: 10.1016/j.jconrel.2024.12.069, PMID: 39742920

[6] van Harten AC Visser PJ Pijnenburg YA Teunissen CE Blankenstein MA Scheltens P. Cerebrospinal fluid Aβ42 is the best predictor of clinical progression in patients with subjective complaints. Alzheimers Dement. (2013) 9:481–7. doi: 10.1016/j.jalz.2012.08.004, PMID: 23232269

[7] Lee EE Hong S Martin AS Eyler LT Jeste DV. Inflammation in schizophrenia: cytokine levels and their relationships to demographic and clinical variables. Am J Geriatr Psychiatry. (2017) 25:50–61. doi: 10.1016/j.jagp.2016.09.009, PMID: 27840055 PMC5164855

[8] Wang K Li X Wang X Hommel B Xia X Qiu J. *In vivo* analyses reveal hippocampal subfield volume reductions in adolescents with schizophrenia, but not with major depressive disorder. J Psychiatr Res. (2023) 165:56–63. doi: 10.1016/j.jpsychires.2023.07.012, PMID: 37459779

[9] Scott J Crouse JJ Medland SE Mitchell BL Gillespie NA Martin NG. Polygenic risk scores and help-seeking behaviour in young people with recent onset of mood and psychotic disorders. J Affect Disord. (2025) 372:40–7. doi: 10.1016/j.jad.2024.11.067, PMID: 39615756

[10] Bzdok D Meyer-Lindenberg A. Machine learning for precision psychiatry: opportunities and challenges. Biol Psychiatry Cognit Neurosci Neuroimaging. (2018) 3:223–30. doi: 10.1016/j.bpsc.2017.11.007, PMID: 29486863

[11] Wu S Jiang Q Wang J Wu D Ren Y. Immune-related gene characterization and biological mechanisms in major depressive disorder revealed based on transcriptomics and network pharmacology. Front Psychiatry. (2024) 15:1485957. doi: 10.3389/fpsyt.2024.1485957, PMID: 39713769 PMC11659238

[12] Feczko E Balba NM Miranda-Dominguez O Cordova M Karalunas SL Irwin L. Subtyping cognitive profiles in Autism Spectrum Disorder using a Functional Random Forest algorithm. Neuroimage. (2018) 172:674–88. doi: 10.1016/j.neuroimage.2017.12.044, PMID: 29274502 PMC5969914

[13] Ni T Sun Y Li Z Tan T Han W Li M. Integrated transcriptome analysis reveals novel molecular signatures for schizophrenia characterization. Adv Sci (Weinh). (2025) 12:e2407628. doi: 10.1002/advs.202407628, PMID: 39564883 PMC11727269

[14] Xie H Wang J Zhao Q. Identification of potential metabolic biomarkers and immune cell infiltration for metabolic associated steatohepatitis by bioinformatics analysis and machine learning. Sci Rep. (2025) 15:16596. doi: 10.1038/s41598-025-86397-x, PMID: 40360670 PMC12075577

[15] Wenhui L Nan W Jiayi H Ye X Chunyu H Zhongzhou L. Prognostic analysis and identification of M7G immune-related genes in lung squamous cell carcinoma. Front Immunol. (2025) 16:1515838. doi: 10.3389/fimmu.2025.1515838, PMID: 40098956 PMC11911325

[16] Subramanian A Tamayo P Mootha VK Mukherjee S Ebert BL Gillette MA. Gene set enrichment analysis: a knowledge-based approach for interpreting genome-wide expression profile. Proc Natl Acad Sci U.S.A. (2005) 102:15545–50. doi: 10.1073/pnas.0506580102, PMID: 16199517 PMC1239896

[17] Barbie DA Tamayo P Boehm JS Kim SY Moody SE Dunn IF. Systematic RNA interference reveals that oncogenic KRAS-driven cancers require TBK1. Nature. (2009) 462:108–12. doi: 10.1038/nature08460, PMID: 19847166 PMC2783335

[18] van Os J Kapur S. Schizophrenia. Lancet. (2009) 374:635–45. doi: 10.1016/S0140-6736(09)60995-8, PMID: 19700006

[19] Mistry M Gillis J Pavlidis P. Genome-wide expression profiling of schizophrenia using a large combined cohort. Mol Psychiatry. (2013) 18:215–25. doi: 10.1038/mp.2011.172, PMID: 22212594 PMC3323740

[20] Purcell SM Wray NR Stone JL Visscher PM O’Donovan MC Sullivan PF. Common polygenic variation contributes to risk of schizophrenia and bipolar disorder. Nature. (2009) 460:748–52. doi: 10.1038/nature08185, PMID: 19571811 PMC3912837

[21] Shamir A Yitzhaky A Segev A Haroutunian V Katsel P Hertzberg L. Up-regulation of S100 gene family in brain samples of a subgroup of individuals with schizophrenia: meta-analysis. Neuromolecular Med. (2023) 25:388–401. doi: 10.1007/s12017-023-08743-4, PMID: 37005977

[22] Chen Y Ouyang Y Li Z Wang X Ma J. S100A8 and S100A9 in cancer. Biochim Biophys Acta Rev Cancer. (2023) 1878:188891. doi: 10.1016/j.bbcan.2023.188891, PMID: 37001615

[23] Boucher J Gilbert C Bose S Tessier PA. S100A9: the unusual suspect connecting viral infection and inflammation. J Immunol. (2024) 212:1523–9. doi: 10.4049/jimmunol.2300640, PMID: 38709994 PMC11076006

[24] Sun Y Wang Z Hou J Shi J Tang Z Wang C. Shuangxinfang prevents S100A9-induced macrophage/microglial inflammation to improve cardiac function and depression-like behavior in rats after acute myocardial infarction. Front Pharmacol. (2022) 13:832590. doi: 10.3389/fphar.2022.832590, PMID: 35814253 PMC9263923

[25] Sun Y Wang Z Wang C Tang Z Zhao H. Psycho-cardiology therapeutic effects of Shuangxinfang in rats with depression-behavior post acute myocardial infarction: Focus on protein S100A9 from proteomics. BioMed Pharmacother. (2021) 144:112303. doi: 10.1016/j.biopha.2021.112303, PMID: 34673424

[26] Qiao CM Tan LL Ma XY Xia YM Li T Li MA. Mechanism of S100A9-mediated astrocyte activation via TLR4/NF-κB in Parkinson's disease. Int Immunopharmacol. (2025) 146:113938. doi: 10.1016/j.intimp.2024.113938, PMID: 39724736

[27] Almeida PGC Nani JV Oses JP Brietzke E Hayashi MAF. Neuroinflammation and glial cell activation in mental disorders. Brain Behav Immun Health. (2020) 2:100034. doi: 10.1016/j.bbih.2019.100034, PMID: 38377429 PMC8474594

[28] Özbay Kurt FG Cicortas BA Balzasch BM De la Torre C Ast V Tavukcuoglu E. S100A9 and HMGB1 orchestrate MDSC-mediated immunosuppression in melanoma through TLR4 signaling. J Immunother Cancer. (2024) 12:e009552. doi: 10.1136/jitc-2024-009552, PMID: 39266214 PMC11409250

[29] Zhao F Hoechst B Duffy A Gamrekelashvili J Fioravanti S Manns MP. S100A9 a new marker for monocytic human myeloid-derived suppressor cells. Immunology. (2012) 136:176–83. doi: 10.1111/j.1365-2567.2012.03566.x, PMID: 22304731 PMC3403264

[30] Portales-Cervantes L Dawod B Marshall JS. Mast cells and natural killer cells-A potentially critical interaction. Viruses. (2019) 11:514. doi: 10.3390/v11060514, PMID: 31167464 PMC6631774

[31] Miller BJ Gassama B Sebastian D Buckley P Mellor A. Meta-analysis of lymphocytes in schizophrenia: clinical status and antipsychotic effects. Biol Psychiatry. (2013) 73:993–9. doi: 10.1016/j.biopsych.2012.09.007, PMID: 23062357 PMC3816144

[32] Li X Hong L Ru M Cai R Meng Y Wang B. S100A8/A9-activated IFNγ(+) NK cells trigger β-cell necroptosis in hepatitis B virus-associated liver cirrhosis. Cell Mol Life Sci. (2024) 81:345. doi: 10.1007/s00018-024-05365-2, PMID: 39133305 PMC11335268

[33] Lood C Stenström M Tydén H Gullstrand B Källberg E Leanderson T. Protein synthesis of the pro-inflammatory S100A8/A9 complex in plasmacytoid dendritic cells and cell surface S100A8/A9 on leukocyte subpopulations in systemic lupus erythematosus. Arthritis Res Ther. (2011) 13:R60. doi: 10.1186/ar3314, PMID: 21492422 PMC3132055

[34] Yang Y Jia W Luo Z Li Y Liu H Fu L. VGLL1 cooperates with TEAD4 to control human trophectoderm lineage specification. Nat Commun. (2024) 15:583. doi: 10.1038/s41467-024-44780-8, PMID: 38233381 PMC10794710

[35] Li Z Zhang J You S Zhang J Zhang Y Akram Z. Pterostilbene upregulates MICA/B via the PI3K/AKT signaling pathway to enhance the capability of natural killer cells to kill cervical cancer cells. Exp Cell Res. (2024) 435:113933. doi: 10.1016/j.yexcr.2024.113933, PMID: 38296018

[36] Nuiyen A Sanguansermsri D Sayasathid J Thatsakorn K Thapmongkol S Ngoenkam J. Nck1 regulates the *in vitro* development of human regulatory T cells through AKT pathway. Clin Exp Immunol. (2025) 219:uxaf011. doi: 10.1093/cei/uxaf011, PMID: 39963999 PMC11923542

[37] Chen S Zhu H Jounaidi Y. Comprehensive snapshots of natural killer cells functions, signaling, molecular mechanisms and clinical utilization. Signal Transduct Target Ther. (2024) 9:302. doi: 10.1038/s41392-024-02005-w, PMID: 39511139 PMC11544004

